# The yin and yang of imaging tumor associated macrophages with PET and MRI

**DOI:** 10.7150/thno.37306

**Published:** 2019-10-15

**Authors:** Sudip Mukherjee, Dominik Sonanini, Andreas Maurer, Heike E. Daldrup-Link

**Affiliations:** 1 Department of Bioengineering, Rice University, Houston, TX-77054, United States; 2 Werner Siemens Imaging Center, Department of Preclinical Imaging and Radiopharmacy, University Hospital Tuebingen, Eberhard Karls University, Tuebingen, Germany; 3 Department of Internal Medicine VIII, University Hospital Tuebingen, Eberhard Karls University, Tuebingen, Germany; 4 Department of Radiology, Stanford University, 725 Welch Rd Stanford, CA 94305-5614

**Keywords:** MRI, PET, Cancer Immunotherapy, Nanoparticles, Radiotracers, Immunotheranostics, Macrophages

## Abstract

Tumor associated macrophages (TAM) are key players in the cancer microenvironment. Molecular imaging modalities such as MRI and PET can be used to track and monitor TAM dynamics in tumors non-invasively, based on specific uptake and quantification of MRI-detectable nanoparticles or PET-detectable radiotracers. Particular molecular signatures can be leveraged to target anti-inflammatory TAM, which support tumor growth, and pro-inflammatory TAM, which suppress tumor growth. In addition, TAM-directed imaging probes can be designed to include immune modulating properties, thereby leading to combined diagnostic and therapeutic (theranostic) effects. In this review, we will discuss the complementary role of TAM-directed radiotracers and iron oxide nanoparticles for monitoring cancer immunotherapies with PET and MRI technologies. In addition, we will outline how TAM-directed imaging and therapy is interdependent and can be connected towards improved clinical outcomes

## Introduction

The immune response to cancer follows two opposite and apparently contradictory principles: some aspects of the tumor immune response can inhibit tumor growth, while others can promote tumor growth 1, 2. Preclinical and clinical evidence showed that an overbearing pro-tumorigenic immune response in malignant tumors significantly promotes tumor growth and metastasis 1, 2. In a pro-tumoral microenvironment, inflammatory M2 tumor associated macrophages (TAM) represent up to 50% of the tumor cell mass 3-6 while immune protective M1 TAM phenotypes are sparse 6-9. Thus, in malignant tumors, the bulk of macrophages in the tumor tissue promote tumor growth. M2 phenotypes augment tumor cell proliferation via elaboration of cytokines, chemokines, proteases and reactive oxygen species 10-12. In addition, M2 TAM enhance angiogenesis via regulating VEGF bioavailability and suppression of protective adaptive immune reactions 13, 14, 15 . Exuberant M2 TAM in breast cancers was strongly associated with poor prognosis, both in animal models and in patients 16-18.

A non-invasive diagnostic test, which can detect and quantify TAM non-invasively, could provide a novel prognostic assay for prediction of tumor progression and poor outcome in cancer patients and could be utilized to lead patients to individualized therapeutic options. Since immune-modulating cancer therapies have been translated to clinical practice, there is an immediate need for imaging tests that can non-invasively quantify macrophage responses in malignant tumors, both from the perspective of patient stratification and monitoring response to novel cancer immunotherapies 7. Positron emission tomography (PET) and magnetic resonance imaging (MRI) are particularly suitable clinical imaging modalities for this purpose since PET radiotracers allow specific targeting with sensitive and quantitative detection of biomarkers, while MRI allows real-time assessment of modulating nanoparticles and imaging of endocytosis without additional radiation dose. We also consider published data on non-PET tracers since they could be adapted to PET by exchange of the isotope.

The purpose of our review article is to discuss the complementary role of TAM-directed radiotracers and iron oxide nanoparticles for monitoring cancer immunotherapies with PET and MRI technologies. In addition, we will outline how TAM-directed imaging and therapy is interdependent and can be interconnected towards improved clinical outcomes.

## Macrophage targeting radiopharmaceuticals

Several radiotracers have been developed to target macrophages *in vivo* in the preclinical and clinical setting. In the past years, macrophage-targeted radiotracers have been mainly used to investigate inflammation-associated diseases including neuroinflammatory, rheumatoid and infectious diseases 19, 20. More recently, a growing number of studies suggest the value of macrophage-specific radiotracers in the field of cancer diagnosis and therapy, which will be discussed in this section.

The high sensitivity of nuclear imaging, particularly PET, enables very low tracer doses, minimizing the effect on the biological system. However, radiopharmaceuticals can also affect macrophages, with the extent and outcome depending on the isotope used. Due to the plasticity of macrophage polarization markers, with overlapping and non-macrophage-restricted expression, it is challenging to develop specific molecular imaging tracers for specific TAM phenotypes. It is also important to recognize that the tracer material on its own can alter macrophage polarization. To evaluate tumor response to TAM modulating cancer treatments, it may be helpful to develop radiotracers that can distinguish between pro-tumorigenic and anti-tumorigenic macrophages.

The macrophage mannose receptor (MMR) CD206, the macrophage scavenger receptor CD163 and the folate receptor beta (FR-β) are the most distinctive surface markers for M2 differentiation *ex vivo* and therefore a primary subject of tracer development 21. Targeting certain functional features such as phagocytic activity and antigen presentation is another focus and subject of endocytosis-associated and MHC class II (MHC-II) specific radiotracers. However, those functional markers may also be represented on other antigen-presenting cells and not specific for macrophages.

**Figure 1:**
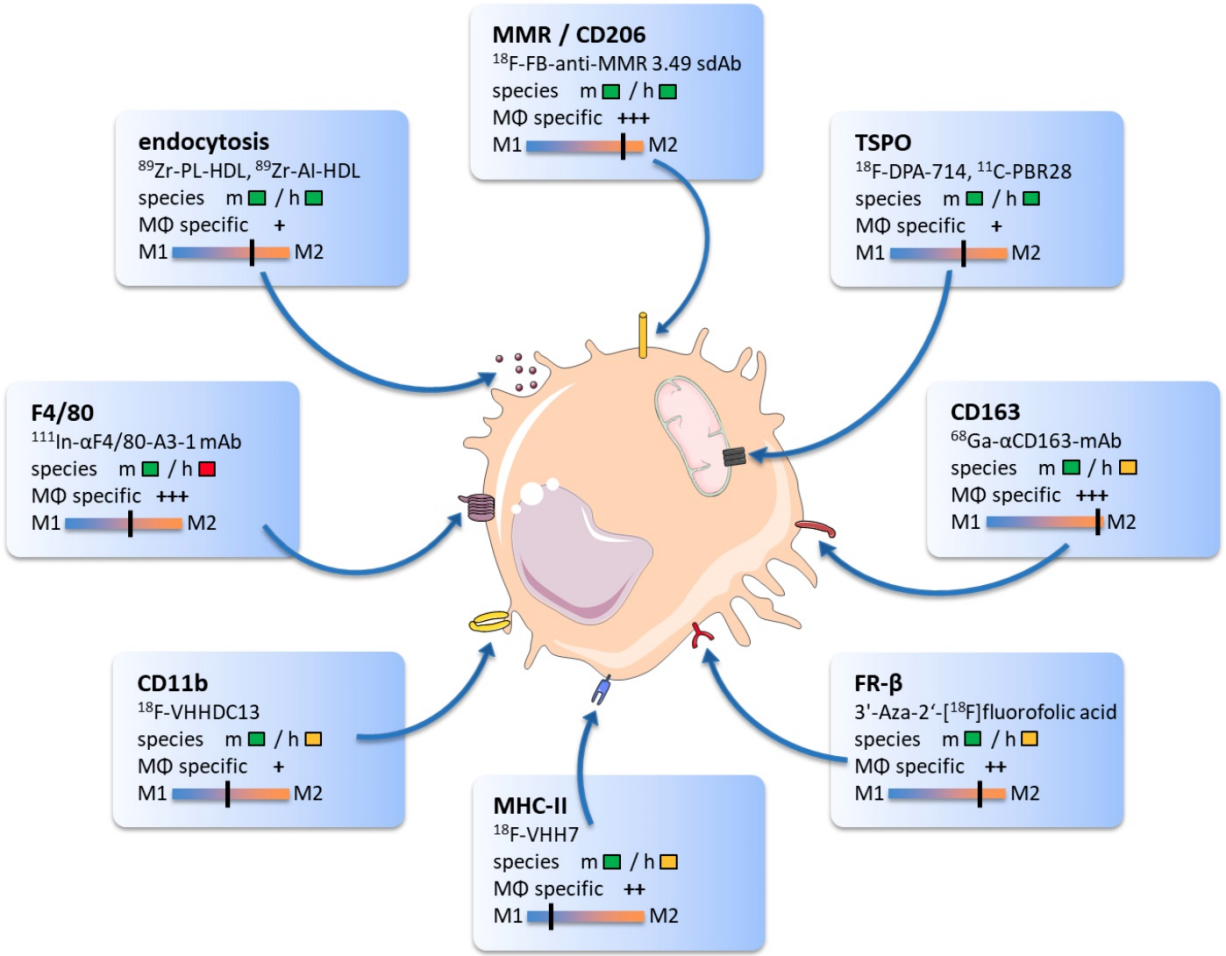
The graphic comprises promising targets for TAM imaging and related radiotracers. Target reactivity for mouse (m) and human (h) species is classified as fully developed tracer ( 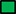 ), tracer development possible ( 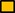 ) and no species specific target expression ( 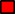 ). Tracer specificity for selective targeting of macrophages (MΦ) is listed for each target ranging from low (+) to high (+++) macrophage specific signal. Target specificity towards M1 or M2 phenotype was estimated based on existing literature and marked as black line.

The following overview will highlight promising TAM biomarkers, their target specificity, and potential value for immunotherapy monitoring. We will provide examples of radiotracers that bind to particular proteins and discuss their ability to modify antitumoral therapies. The core findings of this section and related imaging examples are illustrated in **Figure 1**.

### Translocator protein (TSPO)

The 18 kDa mitochondrial translocator protein (TSPO) has first been discovered in 1977 as an alternative binding site for diazepam in the kidneys 22. TSPO is localized on the outer membrane of mitochondria and is involved in several critical cellular functions such as steroid synthesis, apoptosis and cell proliferation 23. TSPO is primarily expressed in activated microglia, astrocytes, and infiltrating macrophages and is therefore a promising target for imaging inflammatory diseases 24.

There is evidence that TSPO is preferentially upregulated in M2 macrophages, but TSPO was also found on M0 and M1 macrophages, as well as other immune, stromal and endothelial cells 25. Further, TSPO is upregulated in different cancer cells including brain, gastrointestinal, breast, and prostate tumors, potentially hampering its use for detection of TAM 26.

The use of TSPO-specific radiotracers has first been described in different neuroinflammatory diseases and brain injury 27. While the first-generation TSPO radiotracer PK11195 suffers from a low signal-to-noise ratio due to its low brain permeability, nonspecific and plasma protein binding, second generation tracers are limited by overrepresentation of endothelial uptake in the blood-brain barrier and influence of the genetic background on the binding 28.^18^F-GE-180, a high-affinity third-generation radiofluorinated TSPO receptor ligand, demonstrated higher target-to-background ratios and allowed detection of gliomas in patients with a high tumor-to-background ratio 29. However, it has not been elucidated to what extent the radiotracer signal in the tumor tissue was due to ^18^F-GE-180 uptake by glioma cells or TAM. More specific macrophage imaging has been shown in non-cancer mouse models of atherosclerosis by ^18^F-GE-180 30, cardiac myocytes transplantation by ^18^F-DPA-714 31, and tuberculosis using ^125^I-DPA-713 32.

**Figure 2:**
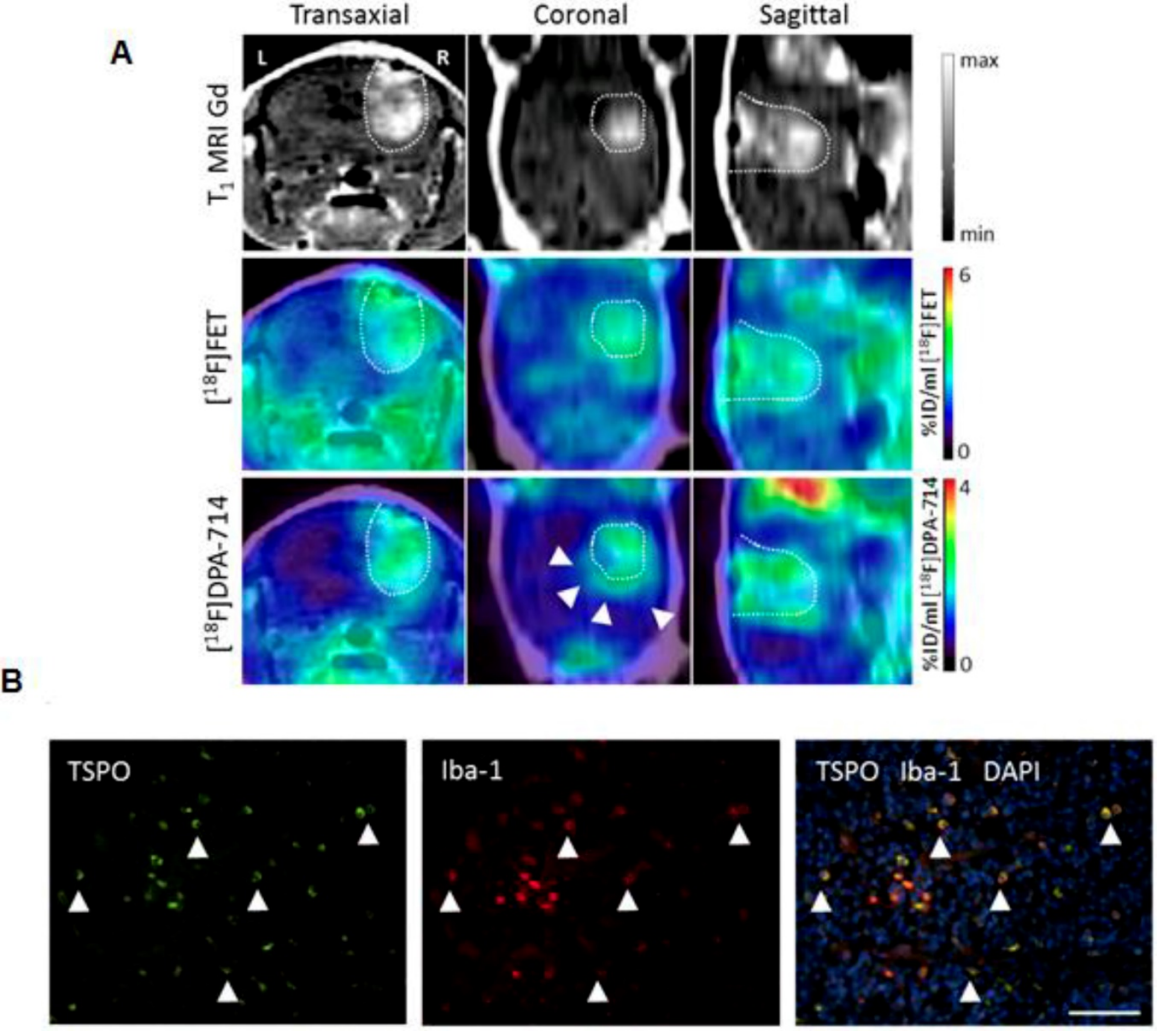
Multi-tracer and multi-modality imaging for the characterization of the glioma immune microenvironment. (A) Gadolinium enhanced T1 weighted (T1 MRI Gd) of a human glioma model. PET-MR images for [^18^F]fluoroethyl tyrosine and the TSPO tracer [^18^F]DPA-714 30 provide complementary information of heterogeneous glioma tissues (arrows). (B) Immunofluorescence analysis reveals tumor-associated macrophages/microglia (Iba-1, red) as important source of TSPO (green) in this model. Scale bar: 50 µm. Image courtesy of: B. Zinnhardt, C. Foray, C. Barca, O. Grauer, M. Schäfers and A. H. Jacobs, unpublished.

As one of the few TAM-focusing studies, Zinnhardt and colleagues identified specific compartments along mouse glioma margins with enhanced TSPO-specific ^18^F-DPA-714 uptake (**Figure 2**). Glioma-associated microglia/macrophages were identified as TSPO source 33. In another preclinical study, Lanfranca et al. were able to track macrophage infiltration in a mouse model of pancreatic cancer with the PET tracer ^11^C-PBR28 34. Tracer uptake clearly correlated with histological macrophage tumor infiltration and specificity was proven in CD11b-deficient mice. However, the authors did not further analyze the subtypes of macrophages. TSPO is a sensitive marker for macrophages and TSPO tracers are clinically translatable 29, however they have not been shown yet to target M2 TAM specifically.

### Macrophage mannose receptor (MMR, CD206)

The macrophage mannose receptor (CD206) is an endocytic carbohydrate-binding receptor expressed by selected populations of macrophages, dendritic cells and nonvascular endothelium. The roles attributed to this receptor include receptor-mediated endocytosis for MHC class II antigen presentation as well as modulation of cell activation and trafficking 35, 36. MMR is preferentially expressed on M2 macrophages and has shown predictive value for cancer progression in different cancer types 37.

Mannose receptor targeting radiotracers were previously evaluated in mouse models of atherosclerosis 38, 39. γ-Tilmanocept (Lymphoseek®), a 18 kDa mannose-decorated dextran labeled with ^99m^Tc, was approved by the FDA for cancer sentinel lymph node detection 40, 41. However, mannose and its analogues are not specific for CD206. Binding of other mannose receptors, such as CD209 expressed in the skin as well as intestinal and genital mucosa was reported 42, 43.

**Figure 3:**
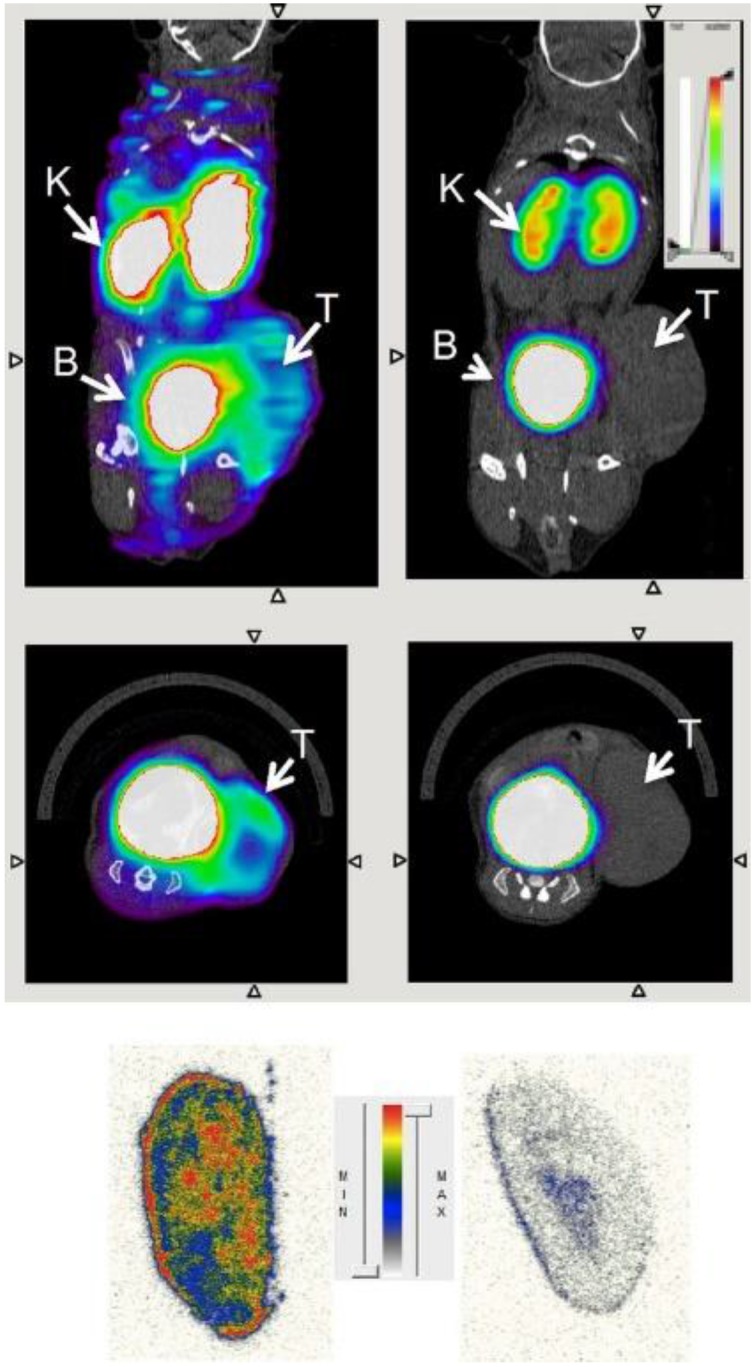
Transverse (upper row) and coronal (middle row) PET/CT images of wild-type (left) and MMR-deficient (right) 3LL-R tumor-bearing mice 3 h after injection of ^18^F-FB-anti-MMR3.49. PET signals are encoded in color scale, CT image in gray scale. Arrows point to tumor (T), kidney (K), and bladder (B). Autoradiography performed on slices from 3LL-R tumors grown in WT (left) vs. MMR-deficient (right) mice are shown in the bottom row. max = maximum; min = minimum. Reproduced after permission from 45. Copyright © 2015 Society of Nuclear Medicine and Molecular Imaging.

To enable more specific targeting, Zhang and colleagues used a radiolabeled anti-CD206 monoclonal antibody for non-invasive imaging of M2 macrophages, which served as an early biomarker for tumor relapse and lymph node metastasis in a murine breast cancer mouse model 44. The groups of Devoogdt, Ginderachter and Caveliers generated single domain antibody fragments derived from camelids with high-affinity binding to CD206. These nanobodies with circulation and tissue penetration characteristics optimized for imaging were labeled with ^99m^Tc and ^18^F. The researchers demonstrated that macrophages in the tumor stroma were specifically targeted by the CD206 nanobodies 45, 46. *In vivo* PET/CT images of ^18^F are shown in **Figure 3**. Notably, the authors selected cross-species reactive nanobodies, which bind both to the mouse and human CD206 homologue with high affinity. This strategy allows direct translation from preclinical evaluation to first clinical studies using the same compound. Hence, CD206 targeting nanobodies are promising candidates for clinical imaging of M2-polarized macrophages.

### Folate receptor β (FR-ß)

Folate receptors (FR) are glycosylphosphatidyl (GPI)-anchored plasma membrane proteins that bind folate and folic acids. Folate plays a complex role in the prevention and progression of cancer: reduced folate taken up by normal cells can prevent tumor development by supporting DNA repair of normal cells 47. However, pre-neoplastic cells upregulate FR as their major and distinct route for endocytosis of non-reduced folate into the cell 47-50. FR density on tumor cells increased as the cancer progressed and was associated with poor outcome in women with breast cancer 49-53.

There are several FR isoforms (α, β, and γ). The α isoform is over-expressed on the membrane surface of cancer cells and serves as a promising target for molecular therapies 54. In contrast, the folate receptor beta (FR-β) is mainly restricted to myeloid cells. It is expressed by tumor-associated macrophages and is a marker for M-CSF induced M2 anti-inflammatory macrophages 55.

FR-β as a macrophage-specific imaging target has been investigated preclinically in rheumatoid arthritis 56 and in clinical studies of patients suffering from atherosclerosis 57 or chronic obstructive pulmonary disease (NCT03494114). Moreover, we identified two single-photon emission computerized tomography (SPECT) studies using radiolabeled folate 58, 59 and one PET study with 3′-aza-2′-^18^F-fluorofolic acid in tumors 60. However, the authors used FR-α expressing tumors, and folate analogues are not FR-ß selective. Thus, it could not be differentiated whether the tumor uptake of the tracers was derived specifically by the macrophages or by the tumor cells. It has further been shown that FR-ß is also expressed on certain human cancer cells, which might limit clinical applicability as a macrophage specific tracer 61.

### Macrophage scavenger receptor CD163

The macrophage scavenger receptor CD163 is a high-affinity binder to the hemoglobin-haptoglobin complex and functions as a sensor for bacteria 62, 63. In contrast to the aforementioned targets, CD163 is thought to be restricted to the monocytic-macrophage lineage, which makes it a very attractive imaging biomarker for these cells 64, 65. CD163 is seen as one of the most reliable markers for M2-polarized macrophages and a proven predictive marker for tumorigenesis 21, 66.

PET imaging of a ^68^Ga labeled antibody for CD163 has been performed in rats with collagen-induced arthritis 67, but to our knowledge not yet in cancer models. Thus, CD163 is a very promising target for imaging of M2 macrophages but there is a lack of cancer specific evaluation and validation. Additionally, a soluble form of CD163 has been identified that is shed by the protease ADAM17 in humans but not in mice 68. This has to be considered before its application towards clinical translation.

### Active endocytosis

Macrophages are major phagocytic cells that engulf pathogens and present their peptide fragments to helper T cells. Apart from pathogens, nanoparticles and liposomes are preferentially phagocytosed by macrophages, monocytes, dendritic cells, and neutrophils 69.

**Figure 4:**
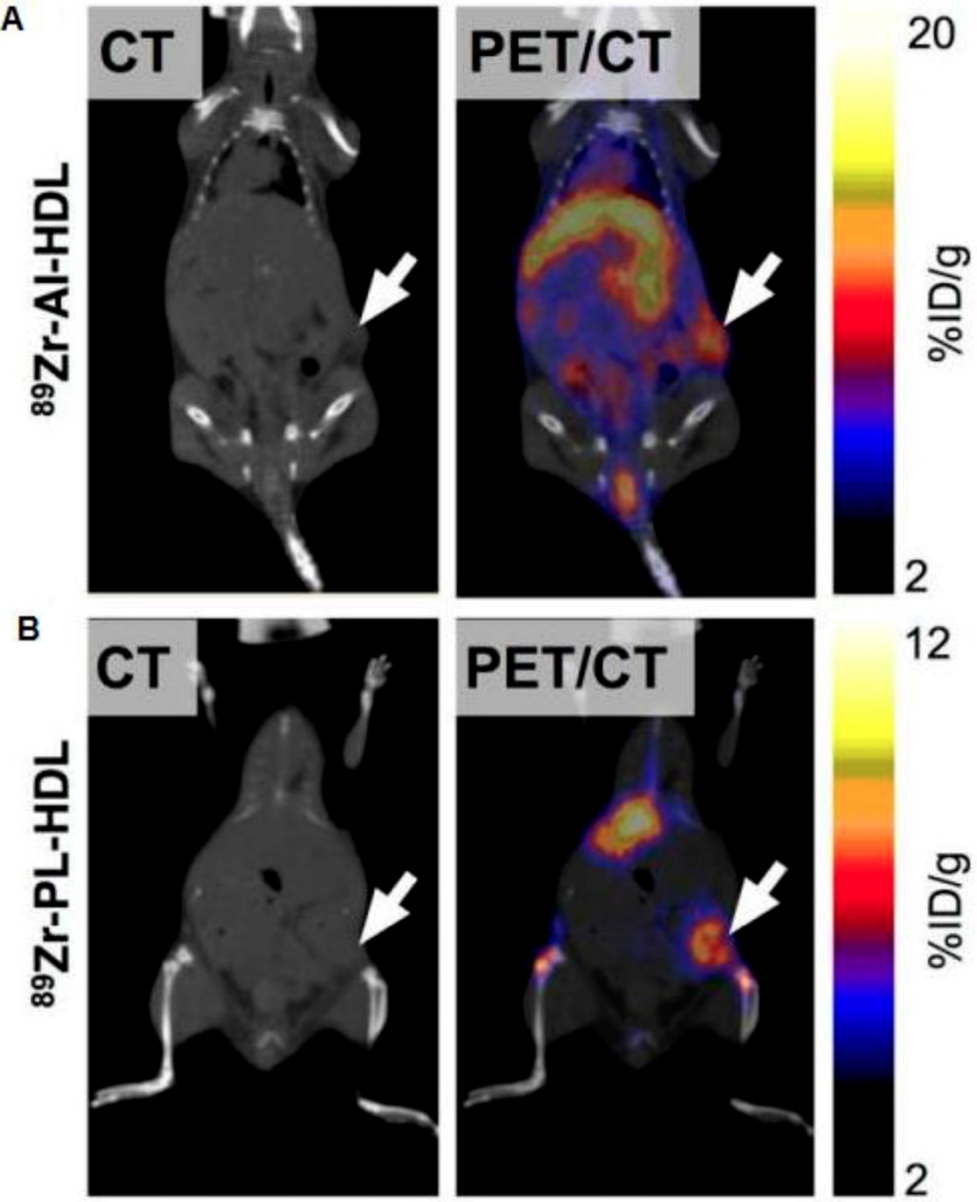
Visualisation of ^89^Zr-HDL nanotracer accumulation in tumor tissues by *in vivo* PET imaging. CT and PET/CT fusion sections of ^89^Zr-AI-HDL (A) and ^89^Zr-PL-HDL (B) obtained 24 h after injection in mice bearing orthotopic 4T1 tumors (indicated by arrows). Reproduced after permission from 71. Copyright © 2015 Society of Nuclear Medicine and Molecular Imaging.

Macrophage-directed nanoparticles have been preferentially developed for MRI imaging. This is because macrophage phagocytosis takes several hours. To image tumor associated macrophages with MRI, we inject nanoparticles intravenously on day 1, wait for nanoparticle tumor perfusion, extravasation and phagocytosis and then image nanoparticles in TAM on day 2. Corresponding imaging techniques with PET or SPECT would require radiotracers with long half-lives such as 64Cu and 89Zr and hence, would be associated with high radiation exposure. To note, dextranated and DTPA-modified magneto-fluorescent 20-nm nanoparticle was labeled with the PET isotope ^64^Cu to allow preclinical PET/CT Imaging of macrophages in inflammatory atherosclerosis 70. Pérez-Medina and colleagues further showed that ^89^Zr-labeled high-density lipoprotein nanoparticles composed of phospholipids and apolipoprotein A-I preferentially targeted TAM 71. Respective PET/CT images are shown in **Figure 4.**

### Other targets

EGF-like module-containing mucin-like hormone receptor-like 1 (EMR1), better known as F4/80, is a mouse-specific pan macrophage marker widely used as target in flow cytometry and fluorescence microscopy. Terry et al. developed an ^111^In-anti-F4/80 antibody to detect macrophages in spleen and tumors 72. However, the characteristic F40/80 macrophage surface antigen is mouse specific. Its human homologue EMR1 is an eosinophil-specific marker and not suitable for macrophage imaging in the clinics 73.

Nanobodies specific for mouse class II MHC (MHC II) and CD11b were created and labeled with ^18^F and ^64^Cu by the group of Ploegh to detect myeloid cells in tumors and lymphoid organs. CD11b is a pan monocytic marker whereas MHC II is expressed on antigen presenting cells such as dendritic cells, B cells and M1 macrophages. The authors were able to visualize myeloid cell infiltration in a syngeneic and xenograft melanoma mouse model 74.

Notably, in contrast to all aforementioned tracers, the mouse MHC II radiotracer targets preferentially M1 macrophages. However, MHC II is expressed on a variety of different cell populations such as B cells and dendritic cells, which makes uptake values difficult to interpret. Furthermore, clinical translation is inherently challenging for antibody-based tracers since they often lack cross-reactivity between species and often need to be developed specifically for the species in question. In the case of antibodies for MHC the polygenic and polymorphic nature of the genetic locus further complicates development of a generally applicable probe.

The landscape of further potential biomarkers and tracers for macrophages goes far beyond the scope of this review. Substantial work on macrophage imaging has been done in the field of inflammation and atherosclerosis. Amongst others, SLC18B1 75, iNOS 76, system x_c_^-^ 77, somatostatin receptor 78, the chemokine receptor type (CXCR4) 79, and cysteine cathepsins 80, 81 have been successfully explored for macrophage imaging (including optical imaging) but either lack suitable PET tracers or await validation for cancer-associated macrophages. An extensive list of established radiotracers in the preclinical and clinical setting with focus on inflammatory diseases is available in the review of Jiemy and colleagues 19.

### Image-guided macrophage-targeting therapies

Novel immunotherapies can either suppress tumor promoting M2 TAM or activate M1 TAM to attack and kill tumor cells. Tumor growth and metastasis formation can be decreased by TAM depletion, by inhibition of TAM recruitment and pro-tumoral function or by reprogramming TAMs into a pro-inflammatory M1 phenotype 82, 83. TAM imaging can help to stratify patients with TAM-rich tumors to TAM-modulating therapies and monitor response to these therapies.

Macrophage targeting antibodies and other molecules are also used for targeted drug delivery. Examples are saporin toxin antibodies that bind to the anti-scavenger receptor A (CD204) 84 and FR-β binding immunotoxins conjugated to *Pseudomonas* exotoxin 85. Especially the M2-specific targets such as CD206 and CD163 are promising candidates for specific modulation, inhibition or depletion of macrophages 86-88. Receptor quantification and dose estimation to targeted drug and radionuclide delivery by PET using the respective radiotracers would enable treatment stratification and therapy response prediction 89. This also accounts for PET imaging of the anti-phagocytic CD47 molecule expressed on cancer cells to estimate outcome of SIRPα/CD47-blocking antibody therapies 90, 91.

There is emerging evidence of radiation to have impact on antitumoral immune responses by release of tumor antigens, inflammatory signals and immune cell infiltration 92, 93. Macrophage polarization can be influenced by external beam radiation. However, this effect seems to be dose-dependent and might also enhance invasive capability of tumor cells 94, 95.

Radiation dose of PET tracers are usually far below the therapeutic window, but therapeutic radionuclides such as ^177^Lu or ^212^Bis bound to target-specific pharmaceuticals can deliver relatively high doses to the target cells 96, 97. First preclinical studies have shown synergistic effects of a vla-4-targeted radionuclide therapy and checkpoint blockade on immune cell infiltration and therapy response in a melanoma mouse model 98. A clinical phase 1b study is underway to evaluate ^177^Lu-PSMA-617 and the immune checkpoint blocking antibody Pembrolizumab (NCT03805594).

In a theranostic setting, therapeutic radionuclides targeting CD206 or CD163 could reduce the number of pro-tumorigenic M2 TAMs, or potentially differentiate M2 TAMs into a M1 polarized phenotype. Although there is no data showing direct radiation effects on TAMs by radiopharmaceuticals, we believe this opens a promising new field in nuclear medicine.

Despite the ongoing developments in cancer treatment especially in the field of immune oncology, it is still evident that the majority of patients suffer from primary or acquired therapy resistance often mediated by immunosuppressive macrophages. The aforementioned therapy approaches emphasize the need of dedicated imaging techniques to detect and quantify different TAM populations with the goal to improve therapy outcome of cancer patients.

## Monitoring cancer immunotherapy with iron oxide nanoparticles

Molecular imaging techniques for cancer imaging have largely focused on imaging cancer cells, cancer cell surface markers, tumor angiogenesis or the extracellular matrix 99-101. However, the inflammatory component of the cancer microenvironment has not been a major target of imaging technologies for magnetic resonance imaging (MRI) thus far. Inflammatory macrophages have been imaged with nanoparticle-enhanced MRI in other inflammatory conditions, such as atherosclerosis and arthritis 102-105. Inflammatory macrophages in malignant tumors have been targeted with preclinical imaging probes for combined fluorescence and MR imaging 106, ^89^Zr-labeled reconstituted high-density lipoprotein (rHDL) nanoparticles 71 and ^64^Cu-labeled mannosylated liposomes (MAN-LIPs) for PET imaging 107, a multimodality probe for fluorescence imaging, MRI and intravital microscopy 108, ^99m^Tc-labeled anti-MMR (macrophage mannose receptor) nanobodies for single-photon emission computed tomography (SPECT)/micro-CT 46, Cy7-labeled deoxymannose for near-infrared fluorescence imaging 109 and bacterial magnetic nanoparticles for MRI detection 110. While all of these approaches successfully detected TAM in cancers, they had the limitation that they were not clinically translatable due to *in vivo* toxicities or lack of biodegradation/elimination from the body 108, 111. Several studies previously demonstrated that TAMs can be tracked with MRI contrast agents, such as manganese (Mn) chelates, iron oxide nanoparticles, and fluorine 19 (19F) incorporated perfluorocarbon compounds (PFCs). 112-114. Small molecular gadolinium chelates, which are used for clinical MR imaging applications are not phagocytosed by TAM due to their small size. To achieve TAM targeting, Gd-chelates were conjugated to antibodies, peptides or other targeting moieties including anti-CEA F(ab')_2_, (MAB) RA96, RGDK and ZD2 115-118. However, these TAM-targeted Gd-chelates provided a low sensitivity for MRI detection and were not suitable for clinical translation 119. In addition, it is not clear if Gd-nanoprobes in macrophages are metabolized and eliminated from the body. This is problematic for clinical translation as interstitial Gd-chelates can cause an irreversible soft tissue fibrosis and sclerosis 120, 121. Thus far, few studies have focused on clinically translatable imaging technologies that enable the detection of TAM in patients.

Ferumoxytol nanoparticles are the only nanoprobes currently available for clinical imaging of TAM in patients. Other clinically translatable nanoparticle compounds in different stages of clinical development include ferumoxtran-10 (Sinerem) 121 and Molday Iron Oxide nanoparticles 122. The FDA-approved iron supplement ferumoxytol (Feraheme™) is currently the only nanoparticle compound that is FDA approved and readily clinically available as an imaging agent via an “off label” use. Ferumoxytol is composed of iron oxide nanoparticles used for intravenous treatment of patients with iron deficiency 123. However, ferumoxytol nanoparticles also provide measurable signal changes on MRI and can therefore be used as an MR contrast agent 124. Intravenously injected ferumoxytol nanoparticles initially distribute in the blood pool due to their large size. Unlike larger nanoparticles (>50 nm), ferumoxytol nanoparticles transiently escape phagocytosis in liver, spleen and bone marrow, which leads to prolonged blood half-life and leaking across hyperpermeable tumor microvessels. The nanoparticles slowly accumulate in the interstitium of malignant tumors, where they are phagocytosed by TAM. This phagocytosis is a slow process, requiring delayed imaging for macrophage depiction at 24 hours after iron oxide injection 102-105. At 24 hours postcontrast, experimental data revealed a specific cellular uptake and MR enhancement of ferumoxytol in TAM isolated from adenocarcinomas 125, 126. No or minimal ferumoxytol uptake was noted in cancer cells 125. The differential high ferumoxytol uptake by TAM and low or absent uptake by cancer cells is the basis for successful TAM imaging. Several studies in animal models and patients have shown that ferumoxytol nanoparticles are compartmentalized in TAMs at 24 hours post injection (p.i.). On these 24 hour delayed scans, the negative (dark) tumor enhancement on T2-weighted MR imaging studies correlated with TAM distribution on histopathology 125. Cellular uptake of iron oxide nanoparticles led to a decreasing T1-signal effect, but persistent T2-signal effect on MR images 127. This “decoupling” of T1- and T2-signal effects on MR images was indicative of intracellular compartmentalization. Recently, this concept has been translated to first-in-human clinical trials and showed that ferumoxytol-MRI can quantify TAM quantities in patients with glioblastoma 128, osteosarcoma and lymphoma 126. Within each tumor group, T2* signal enhancement on MR images correlated significantly with the density of CD68+ and CD163+ TAM (*P* < 0.05) 126, 128.

M2 TAM in breast cancer directly correlated with tumor aggressiveness, and indirectly correlated with clinical outcome 7, 17, 129-131. Preclinical and clinical data have shown that patients whose cancers are heavily infiltrated with TAMs benefit from combining chemotherapy with M2 TAM-antagonist therapeutics. TAM-selective imaging would facilitate identification of this patient population and enable monitoring patients during therapy. In mouse models of breast cancer, blocking TAM infiltration significantly enhanced efficacy of standard-of-care chemotherapy and extended overall survival 83, 132. Daldrup-Link and team showed in preclinical models of breast cancer, that treatment with CSF1 (colony stimulating factor 1) monoclonal antibodies significantly reduced ferumoxytol tumor enhancement on delayed T2-weighted MR images and that this effect correlated with a significant decline in TAM quantities in the tumor tissue on histopathology, as determined by CD68 immuno stains and flow cytometry analyses 125. These data indicated that tumor MR imaging with clinically applicable iron oxide nanoparticles enabled non-invasive quantification of TAM in neoplastic tissue where their presence serves as a novel biomarker for tumor therapy. Since clinical trials of new therapeutic drugs are expensive and take years to complete, the immediate value and impact of this new imaging approach could be immense.

**Figure 5:**
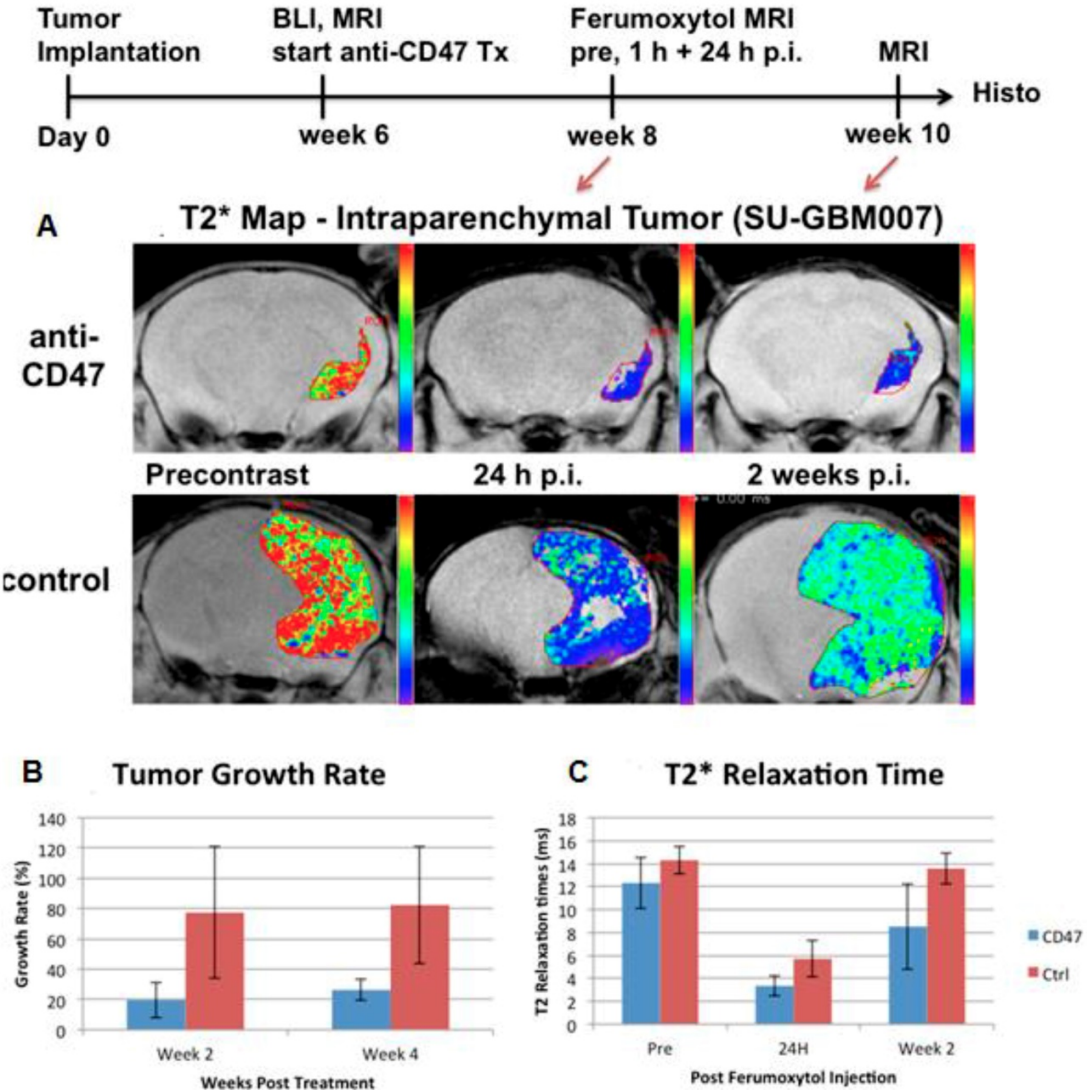
Growth rate and MRI enhancement of human glioblastomas in representative NOD-SCID x RAG g/d dko mice. (A) Representative MR images with superimposed T2* relaxation time maps of anti-CD47 mAb treated tumor (upper row) and sham-treated control (lower row). (B) Corresponding tumor growth rates, calculated as % change in tumor size, and (C) T2* relaxation times of anti-CD47 mAb treated tumors (n=3) and sham-treated controls (n=5), displayed as means and standard deviations. Please note that ferumoxytol-induced T2*-signal enhancement is quantified by shortened T2* relaxation times (shorter T2* time = stronger contrast enhancement).

Of note, more than 90% of macrophages in malignant tumors represent M2 TAM. Therefore, for untreated tumors, there are limited applications of M1 TAM markers. However, cancer immunotherapy can either suppress M2 TAM or activate M1 TAM. A new TAM-directed immunotherapy approach is to reprogram tumor promoting M2 TAM phenotypes into tumor fighting M1 TAM phenotypes 95. Work in the Weissman lab at Stanford has for the first time demonstrated that anti-cancer activity from the innate immune system can be activated via blockade of the immune-suppressive cell surface molecule CD47 expressed on tumor cells 133. CD47 was expressed on the surface of all cancer cells analyzed, and CD47 blockade resulted in activation of anti-cancer activity from macrophages and eradication of tumors in mice 133. This M1-TAM activation lead to increased tumor uptake of non-targeted nanoparticles and this effect could be imaged with MRI: Ferumoxytol-MRI was used to monitor response to anti-CD47 mAb therapy in mouse models of glioblastomas (**Figure 5**) and osteosarcomas 134, as noted by an increasingly negative (dark) nanoparticle enhancement of the tumor tissue compared to pre-treatment scans 134. CD47 mAb cancer immunotherapies have been translated to the clinic and first-in-human Phase I/II clinical trials in patients are currently ongoing. A major side effect of anti-CD47 treatment is anemia. Ferumoxytol is a FDA-approved iron supplement for anemia treatment and might counteract this side effect. CD47 mAb-mediated macrophage polarization towards M1-TAM phenotypes would benefit from specific imaging biomarkers for M1-TAM. However, to our knowledge there are no clinically translatable M1-specific TAM markers for MR imaging to date.

It is important to note that standard chemotherapies and immune modulating therapies do not represent a binary concept. Many standard chemotherapeutic agents have intrinsic immune modulating properties, which are important to understand and consider with planned new combination therapies. For example, Doxorubicin is established for the treatment of osteosarcomas and acts on a common mechanistic pathway with CD47 mAb by inducing immunogenic cell death 135. Doxorubicin induces the expression of calreticulin on the cell surface of sarcoma cells that binds to low-density lipoprotein receptor-related protein 1 (LRP1) and functions as a pro-phagocytic “eat me” signal for TAM 136, 137. We have recently shown that this effect can be visualized with ferumoxytol-MRI. Osteosarcomas in mouse models demonstrated significantly stronger ferumoxytol enhancement and significantly increased TAM quantities after CD47 mAb plus doxorubicin combination therapy compared to CD47 mAb (P = 0.02) and doxorubicin monotherapy (P = 0.001) 186. Since cytotoxic drugs may be either immunostimulatory or immunosuppressive 138, ferumoxytol-MRI might be useful as a new tool to find synergistic drug combinations and recognize antagonistic combinations. While other iron oxide nanoparticles 139, 140 and other metal-doped nanoparticles 141 have been used to label macrophages, they are not clinically translatable and therefore, of uncertain clinical impact.

### Limitations of ferumoxytol-enhanced MRI for TAM detection

Ferumoxytol doses up to 400 mg Fe/kg were non-lethal in rodents 142 and ferumoxytol nanoparticles were generally well tolerated by most patients. However, rare anaphylactic reactions have been described in adult patients 123, 143, 144. Likewise, our initial experience with ferumoxytol administrations did not reveal any side effects. However, due to the risk of rare, but potentially severe allergic or pseudo-allergic reactions, it is important to screen patients for any history of allergies and use, proper, slow iron administration techniques to avoid iron-induced hypotensive reactions.

Makela et al tracked TAMs labeled with iron oxide nanoparticles and perfluorocarbon (PFC) agents with MRI in 4T1 breast tumors 145. A signal loss of the entire tumor was observed after 4 days of iron oxide nanoparticle treatment and a more pronounced signal loss of the tumor periphery was noted at 3 weeks, indicating higher accumulation of TAMs in the tumor periphery at 3 weeks. However, after PFC administration, similar spatial fluorine-19 (^19^F) signal was noted in the tumor center and periphery, indicating the presence of similar TAM quantities in different tumor areas. This study suggested that ^19^F-based TAM tracking methods provide different information compared to the iron-based TAM imaging technology. In another report by Leftin et al, the authors reported that the depiction of TAMs in breast cancer models can be significantly enhanced by focusing on spatial distributions of iron deposits instead of ROI averages 146. Previous studies showed that both M1- and M2-TAMs take up ferumoxytol. Thus, we are not able to discriminate these sub-populations with ferumoxytol. Nanoparticles that are directed to specific surface markers, such as mannose for the M2 subtypes, could provide more specific targeting in the future.

Recently, proliferating macrophages have been identified as an abnormal TAM subpopulation associated with high-grade cancers and increased risk of recurrence 131. We would expect these metabolically active, proliferating TAMs to show marked nanoparticle uptake, while non-activated monocytes may show lesser or no ferumoxytol uptake. This theory will be evaluated by co-localization analysis of anti-dextran and CD68 stains, augmented with other cell-type specific immunostains 131.

### Modulating effects of Iron Oxide Nanoparticles

#### Intrinsic effects of iron oxide nanoparticles on tumor associated macrophages

Iron oxide nanoparticles have gained interest for cancer imaging due to their relatively easy synthesis, small size, high surface to volume ratio, easy functionalization, and multifunctional theranostics capabilities 147-152. The intrinsic effects of magnetic iron oxide nanoparticles on macrophages can occur in the following ways: a) NPs stimulate M1 polarization 153. b) NPs induce ferroptosis 154.

**Figure 6:**
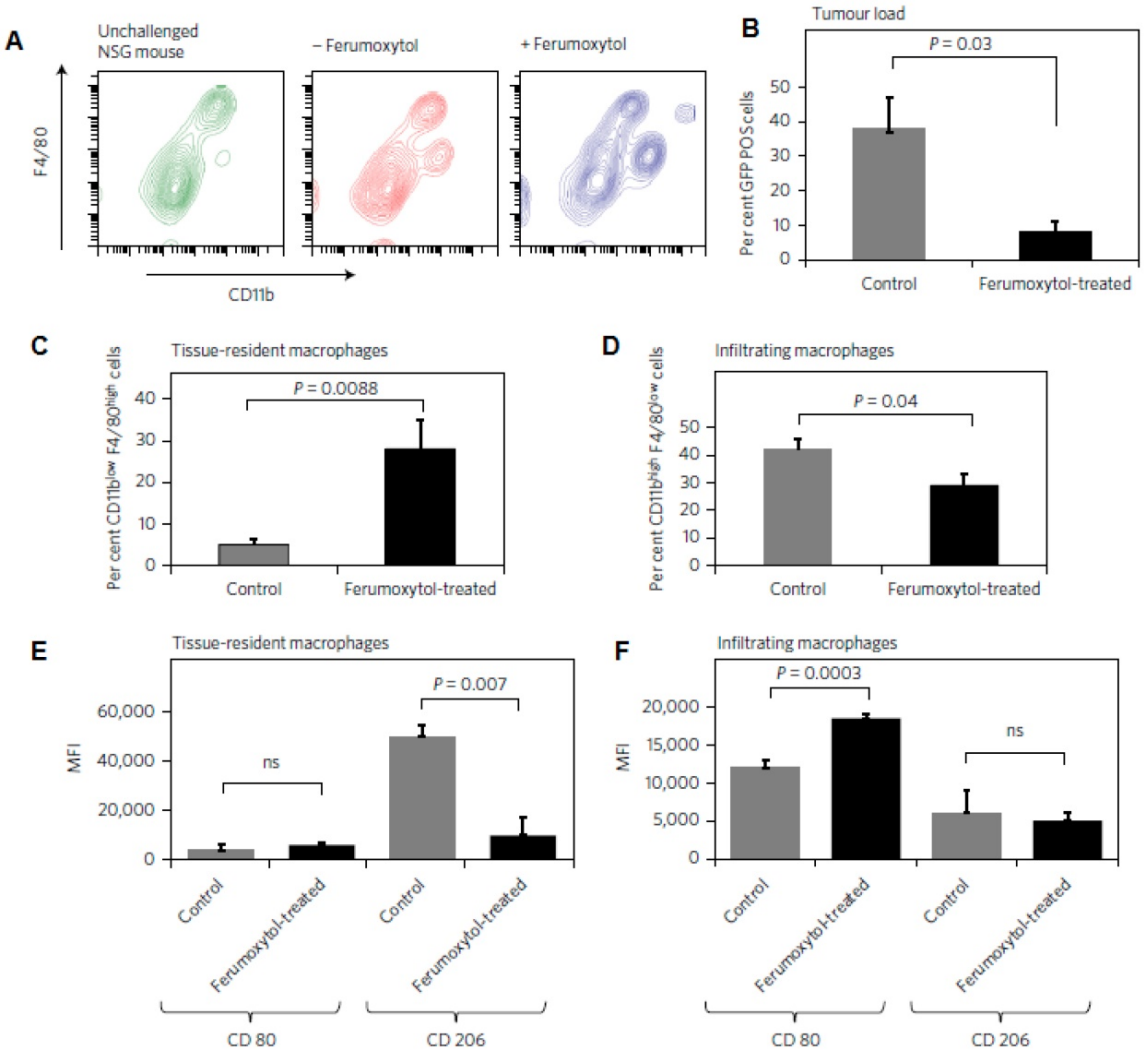
Ferumoxytol alters macrophage polarization in hepatic metastasis *in vivo*. a, Livers of the same mice described in Fig. 6 were further analysed with FACS for infiltrating leukocyte populations. b, Accordingly, the relative number of GFP+ cells within the liver gate (%) was significantly reduced in ferumoxytol-treated livers compared with untreated controls. c, CD11blowF4/80high tissue-resident macrophages were increased in ferumoxytol-treated livers compared to controls and d, CD11bhighF4/80low peripheral-derived macrophages were increased in ferumoxytol-treated livers compared with controls. e,f, The polarization of both tissue-resident and infiltrating macrophages shifted towards the M1 phenotype as measured by CD80 and CD206 markers: median fluorescent intensity ratios (MFI) of M1/M2 associated markers (CD80/CD206) in CD11blowF4/80high tissue-resident macrophages (e) and infiltrating F4/80low CD11bhigh liver macrophages (f) isolated from ferumoxytol-treated livers and untreated controls. All quantitative data are displayed as the mean of seven livers per group ± standard deviation. *P < 0.05, indicates a statistically significant difference (Student's t-test) from untreated controls. Reproduced after permission from 157. Copyright © 2016 NPG.

**Figure 7:**
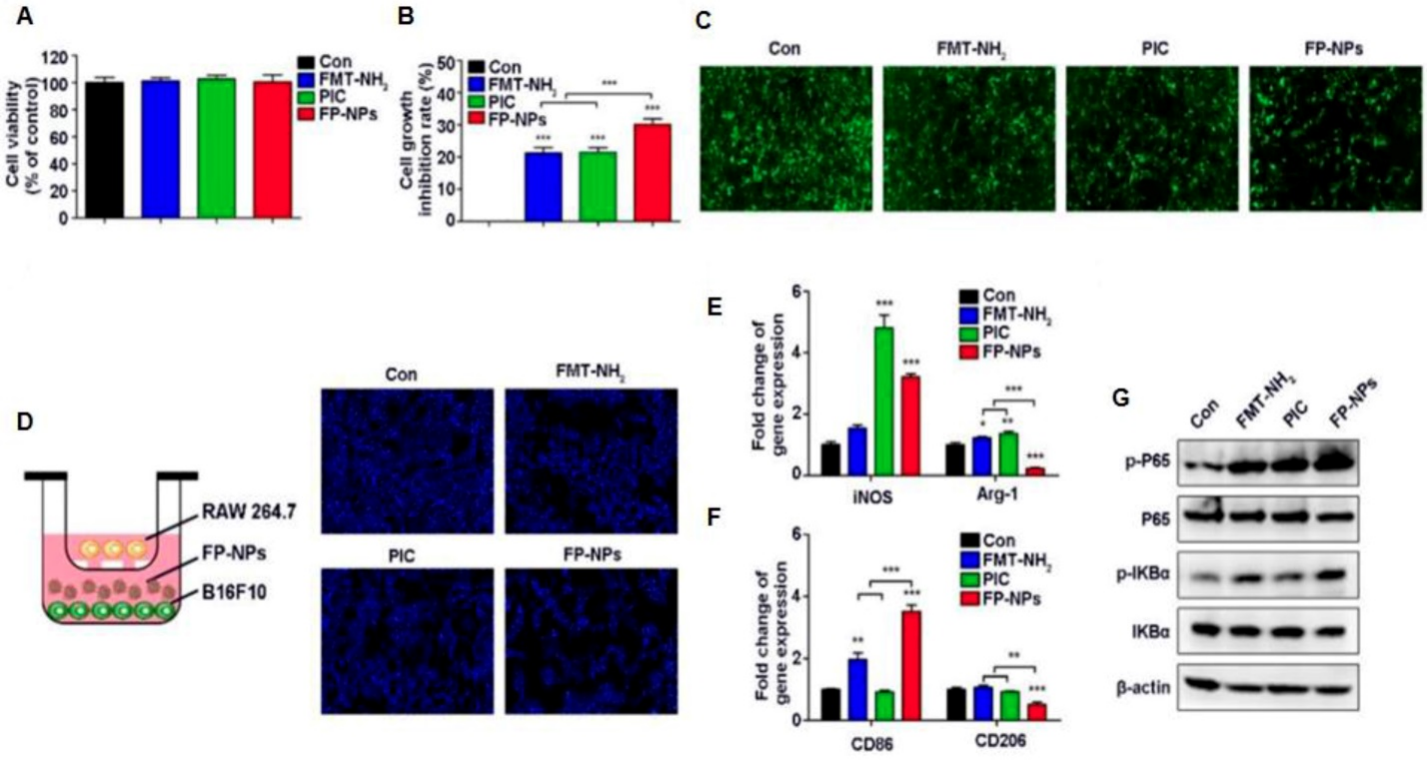
Effect of FP-NPs on tumor cell proliferation and macrophage polarization. (A) B16F10 cells were incubated with FMT-NH2, PIC, or FP-NPs for 48 h and the cell viability was analyzed by CCK-8 assay. (B) B16F10 cells pre-labeled with CFSE co-cultured with RAW 264.7 at a ratio of 2:1 were incubated with FMT-NH2, PIC, or FP-NPs for 48 h and cell proliferation was then analyzed by FCM. (C) GFP-B16F10 cells co-cultured with RAW 264.7 at a ratio of 2:1 were incubated with FMT-NH2, PIC, or FP-NPs for 48 h and the fluorescence intensity of GFP was captured. (D) Left: Schematic diagram of the co-culture. Right: B16F10 cells co-cultured with RAW 264.7 at a ratio of 2:1 were incubated with FMT-NH2, PIC, or FP-NPs in a dual-chamber Transwell system for 48 h and the tumor cells in the lower chamber were stained with DAPI and analyzed by fluorescence microscopy. (E, F) RAW 264.7 were incubated with FMT-NH2, PIC, or FP-NPs for 12 h and the expression of macrophage M1 (iNOS, CD86) and M2 (Arg-1, CD206) related genes was analyzed by qRT-PCR. (G) RAW 264.7 cells were incubated with FMT-NH2, PIC, or FP-NPs for 15 min and the expression of target proteins was analyzed by WB. All representative data are from three independent experiments. Error bars, SD. *P < 0.05, **P < 0.01, ***P < 0.001. FCM: flow cytometry; FMT: ferumoxytol; FP-NPs: FMT-NH2-poly I:C; iNOS: inducible nitric oxide synthase; PIC: poly I:C, polyinosinic-polycytidylic acid. WB: western blotting. Reproduced after permission from 164. Copyright © 2018 Ivyspring International Publisher Pty Ltd.

Iron oxide nanoparticles are presently used as iron replacement therapies clinically 155. Iron exposure can regulate iron transport-related proteins that are associated with macrophage polarization states 156. Recent studies by Daldrup-Link and co-workers showed that ferumoxytol nanoparticles could suppress tumor growth by inducing M1 macrophage polarization in early mammary cancers, and lung cancer metastases in liver and lungs. 157. Tumor cells co-injected with ferumoxytol exhibited a markedly delayed growth rate as compared to tumor cells injected without ferumoxytol 157. Flow cytometry and histopathology showed that the observed tumor growth inhibition was accompanied by increased presence of pro-inflammatory M1 macrophages in the tumor tissues (**Figure 6**) 157. Tumor sections obtained at day 7 after their implantation into experimental mice showed increased presence of CD80+ cells within ferumoxytol-co-implanted tumors compared to controls, apparently representing increased quantities of pro-inflammatory M1 macrophages. Previous *in vitro* studies showed that superparamagnetic iron oxides induce a phenotypic shift in M2 macrophages towards a high CD86+, TNFα positive M1 macrophage subtype 158. In the presence of iron oxide nanoparticles, M1-TAM polarization can induce a Fenton reaction: Activated M1-TAM produce hydrogen peroxide, which reacts with ferrous iron to produce hydroxyl radicals that destroy organic material 159. Cancer cells exposed to hydrogen peroxide and hydroxyl radicals produce oxidized lipids, proteins, and damaged DNA, which can induce cell death. Dying cancer cells produce high levels of reactive oxygen species (ROS), which are released into the extracellular area when the cellular membrane is degraded during cell death. Extracellular H_2_O_2_ serves as chemo-attractant to monocytes and drives monocyte-to-M1 macrophage polarization 160. This continued M1-polarization can create an autocrine feedback loop that maintains the production of TNFα and nitric oxide and thereby, causes continued cancer cell death and tumor growth inhibition. These observations were confirmed in a second animal model of glioblastoma multiforme, where the intrinsic immune-modulatory effect was most effective in early stage tumors, similar to other approaches of cancer immunotherapy 161, 162. Similarly, Zhao et al. recently reported that ferumoxytol nanoparticles induced pro-inflammatory macrophage polarization in aggressive melanoma cancers. Toll-like receptor 3 (TLR3) activation increased the anti-tumor response of immune cells by manipulating cytotoxic T lymphocytes (CTL) and anti-tumor natural killer (NK) cells through the maturation of dendritic cells (DCs) 163, 164. Ferumoxytol combined with a TLR3 agonist, poly (I:C) (PIC), demonstrated synergistic inhibition of tumor growth by shifting macrophages to a tumoricidal phenotype 164. This was also correlated with upregulation of TNF-α and iNOS, with an enhanced NO secretion. In a combination therapy of ferumoxytol with the TLR3 agonist PIC showed superior melanoma regression and anti-metastatic efficacy that is associated within filtration of pro-inflammatory macrophage response and upregulation of pro-inflammatory genes *in vivo* (**Figure 7**) 164. These findings suggest that ferumoxytol nanoparticles hold great potential to macrophage-modulating cancer immunotherapy by inducing the tumor-suppressive macrophage polarization within the tumor microenvironment.

Ferroptosis is a newly illustrated programmed cell death mechanism that takes place via an iron and lipid peroxidation dependent process and is stimulated by glutathione diminution 165, 166. Even though, iron is a promoter of cancer cell proliferation, it plays a crucial role for producing reactive oxygen species (ROS) and lipid peroxidation via the Fenton reaction. Iron metabolism consisting of iron uptake, reflux and storage can also induce ferroptosis, a distinct, iron-dependent type of programmed cell death characterized by the accumulation of lipid peroxides. Some studies suggest that iron-based NPs can induce ferroptosis of cancer cells 154. Iron oxide nanoparticles which can be directed by magnetic fields might be particularly useful for ferroptosis-based cancer therapy. Zhou et al. showed an iron oxide nanoparticle based delivery of linoleic acid hydroperoxide (LAHP) polymers to transport Fe^2+^, generating ROS and ^1^O_2_ that has led to programmed cancer cell death or ferroptosis.^167^. Zhang et al. reported amorphous iron (Fe^0^) nanoparticles (AFeNPs) for ferroptosis-based cancer therapy in a breast cancer model. Mechanistic studies revealed that ionization of the AFeNPs facilitates ferrous ion release in the tumor, which led to H_2_O_2_ and hydroxyl radical generation (•OH or •OOH) 168. Huan et al. demonstrated the assessment of zero-valent iron-based (ZVI NPs) nanotherapeutics for induced cancer cell death or ferroptosis and resensitization strategy based on ferroptosis inducers with minimal side effects to healthy non malignant cells 169.

Apart from ferumoxytol (Feraheme™), various other iron oxide-based nanoparticles including ferucarbotran and ferumoxides have been extensively used as clinically approved MRI contrast agents 170, 171. Treating M2-polarized macrophages with ferucabotran *in vitro* caused an increase in the expression of CD86, ferritin, cathepsin L and M1-like polarization 158. Costa da Silva et al. monitored the accumulation of iron in lung tissues from lung cancer patients 172. Hemolysis-induced iron accumulation was inversely related with CD68 expression on TAM. Iron-containing macrophages demonstrated decreased expression of CD206 (with decrease in tumor size) and increased expression of CD86 and iNOS. Moreover, these macrophages showed an increase in IL-10 and IL-6 secretion indicating an M1-polarizing shift. These results support the functions of iron and hemolytic RBC for the repolarization of TAM to employ an anti-tumor effector function using an adjuvant therapeutic strategy to endorse an anti-cancer immune response.

In a recent published article, Chen et al. showed for the first time the use of iron oxide embedded mesoporous organosilica nanocomposite (IO-LPMONs), for the activation of cytotoxic T cells and polarization of macrophages in tumor immunotherapy 152. These nanoparticles efficiently delivered OVA to DCs, activated DCs, which in turn activated both CD4+ and CD8+ effector antigen specific T cells and ultimately lead to strong anti-tumor effects. Additionally, the IO-LPMONs acted as an immune modulator for the polarization of TAMs from an immunosuppressive M2 to a tumor killing M1 phenotype, inducing efficient tumor apoptosis. This combination of macrophage polarization and T cell activation strategy induced potent anti-tumor effects *in vivo* in EG7-OVA tumor bearing C57BL6 mice. Kodali et al. compared the effects of superparamagnetic iron oxide nanoparticles (SPIONs) and silica nanoparticles (SiNPs) on lung macrophages by measuring differences in gene expressions 173. SPION treatment altered a total of 1029 genes, while SiNPs altered the expression of 67 genes. SPION treatments increased TNF-α secretion and reduced IL-10 secretion from macrophages compared to SiNPs treatments. This showed the stronger M1-polarization effects of SPIONs compared to SiNPs.

SPIONs can also play a role in the generation of reactive oxygen species (ROS) which can induce pro-inflammatory cytokines and interleukins in the tumor microenvironment. Rojas et al. synthesized aminopropyl silane-, di-mercaptosuccinic acid- and amino dextran-coated SPIONs for the generation of ROS in TAM 174. Mulens-Arias et al. reported that the treatment of macrophages by polyethyleneimine (PEI)-coated SPIONs amplified the expressions of ferritin, CD8 and CD86 along with the secretion of IL-12 and IL-10 from macrophages 175.

Alternating magnetic field (AMF) therapy has shown great promise for controlled drug release from iron oxide nanoparticles and for induction of tumor hyperthermia 176. However, the effects of AMF on the macrophage polarization were not well studied. Kang et al. studied the effects of AMF therapy using the treatment of RGD peptide-amino-silica-coated SPIONs on macrophage polarization 177. Following nanoparticle treatment, a low frequency AMF was applied on the mice that demonstrated increased arginase-1 and decreased iNOS in mice macrophages, supporting for M2-like polarization state. Further, high frequency AMF exposure exhibited an M1-like polarization state. Toraya-Brown et al. has demonstrated the application of magnetic hyperthermia by combining iron oxide nanoparticles and AMF 178. The tumor hyperthermia of melanoma tumors activated the dendritic cells (DCs) and CD8+ T cells that promoted a strong resistance against reoccurrence of the melanoma cancer or metastasis. These recent studies thus support that the AMF therapy shows promise as a regulator for macrophage polarization and inducing anti-tumor immune responses using SPIONs therapy.

#### Engineering iron oxide nanoparticles to enhance immune-modulating effects on the cancer microenvironment

Engineering iron oxide nanoparticles to augment or supplement cancer immunotherapies has become an emerging area of research. This section will provide examples of this approach.

Tumor accumulation of T cells for immunotherapy can be enhanced by magnetic navigation of nanoparticle targeted T cells to tumors 179. Mühlberger et al. demonstrated the magnetic navigation of T-cells, which were loaded with SPIONs and immune modulatory drugs 180. They synthesized lauric acid (LA) and albumin coated SPIONs, incubated them with mouse cytotoxic T lymphocytes and attracted tumor accumulation of SPION loaded T cells with an external magnetic field. Perica et al. conjugated Major Histocompatibility Complex-Peptide and co-stimulatory anti-CD28 to paramagnetic iron-dextran coated SPIONs. The resulting artificial antigen-presenting cells (aAPCs) were able to bind to T cell receptors, capturing T-cells in a magnetic column and activating them 181. This resulted in 1000-fold expansion of tumor-specific T cells in one week 181. Zhang et al coated magnetic nanoclusters with azide-engineered leucocyte membranes and T-cell stimuli. The resultant multifunctional aAPCs stimulated CD8+ T cells and enabled *in vivo* tracking of intravenously injected T cells to tumors in mouse models with MRI 182. Nanoscale aAPCs induced regression of tumor growth in an *in vivo* lymphoma model without observable toxicity.

SPIONs have been also used to generate nanovaccines, which train and stimulate the immune system to recognize and combat cancer cells. Cho et al. used multifunctional iron oxide-zinc oxide core-shell nanoparticles to deliver carcinoembryonic antigen into DCs, which induced an immune response and reduced tumor growth and improved mice survival 183. The DCs were transfected *ex vivo* by the nanoparticle-antigen complex, injected into tumor-bearing mice and caused significant tumor antigen T cell response. The nanovaccine could be tracked *in vivo* with MRI. In another recent study, Zhao et al. used SPIONs to deliver Ovalbumin EndoFit (OVA) vaccine 184. In comparison of OVA and SPIONs alone, the OVA-conjugated SPIONs demonstrated significantly increased immune responses and inhibition of tumor growth. The OVA formulated SPIONs showed the activation of immune cells and cytokine production, inducing significant cellular and humoral immune responses.

Shevtsov et al. demonstrated enhanced immunostimulatory activity of SPIONs coated with Hsp70, a heat shock protein. The authors showed the delivery of immunogenic peptides from tumors lysates to DCs by Hsp70-SPIONs that helped to stimulate tumor-specific T cell response and reduce tumor growth by enabling antigen trafficking to APCs 185.

## Conclusion and future perspective

In summary, TAM play a fundamental dualistic role in carcinogenesis, tumor growth and metastasis. Immediately clinically available imaging techniques for *in vivo* detection of TAM in patients include ferumoxytol-enhanced MRI and PET imaging with translocator protein (TSPO)-targeted radiotracers. Future opportunities include the development of TAM biomarkers that are designed to more specifically target pro- and anti-tumoral macrophage phenotypes in the tumor microenvironment. Examples include Mannose-, CD206 and CD-163-targeted radiochemicals for specific imaging of M2-TAM phenotypes.

Furthermore, with the predominant existence of M2-polarized macrophages within the tumor environment, research attempts have almost exclusively focused on the development of M2-directed imaging agents. The emerging interest in macrophage modulating drug development implies the need of M1-targeting imaging techniques as well, for which only very few have been described so far 74.

While significant advances are being made in the development of TAM-specific imaging biomarkers for MRI and PET, future studies can leverage their complementary strengths. On the one hand, the simultaneous application of two complementary imaging agents allows increasing the accuracy and specificity of macrophage targeting. On the other hand, the combination of a macrophage imaging agent (e.g. ferumoxytol-MRI) with further immune cell targeting agents such as the novel T cell specific immunotracers will extend the ability to display complex immune responses by *in vivo* imaging.

Challenges will include the preservation of the high sensitivity of current standard imaging tests for tumor detection while adding specificity. For example, combined tumor detection and TAM quantification could be achieved by combining clinical standard FDG-PET with ferumoxytol MRI or by combining whole body diffusion weighted MRI with TAM-specific PET imaging. More detailed diagnoses of immunotherapy induced changes in M1/M2 TAM compositions of the tumor microenvironment could be achieved by combining ferumoxytol MRI for imaging the total TAM population with radiotracers for imaging M2-TAM. Similarly, the TAM-directed diagnostic probes could be loaded with therapeutic drugs to generate theranostic (combined diagnostic and therapeutic) probes that will enable image-guided, personalized therapies.

## Abbreviations

AMF: alternating magnetic field
CT: computed tomography
EMR1: EGF-like module-containing mucin-like hormone receptor-like 1
FR: folate receptor
IL: interleukin
IONP: iron oxide nanoparticles
IO-LPMON: iron oxide-embedded large-pore mesoporous organosilica nanospheres
M1: macrophages with an inflammatory, antitumoral phenotype
M2: macrophages with an anti-inflammatory, protumoral phenotype
mAb: monoclonal antibody
MHC II: major histocompatibility complex class II
MMR: macrophage mannose receptor
MΦ: macrophage
MRI: magnetic resonance imaging
NP: nanoparticle
PET: positron emission tomography
sdAb: single domain antibody
SiNP: silica nanoparticle
SPECT: single photon emission computed tomography
SPION: superparamagnetic iron oxide nanoparticle
TAM: tumor associated macrophages
TNF-α: tumor necrosis factor alpha
TSPO: mitochondrial translocator protein
